# Chronic disease count and fall-related characteristics among community-dwelling older adults: a cross-sectional study of recurrent falls and fall timing

**DOI:** 10.3389/fpubh.2026.1882689

**Published:** 2026-10-12

**Authors:** Yu Chen, Junliang Zhou, Yuanyuan Cai, Jierong Li

**Affiliations:** 1 Department of Health Management, Neijiang Health Vocational College, Neijiang, Sichuan, China; 2 Department of Orthopedics, Neijiang Hospital of Traditional Chinese Medicine, Neijiang, Sichuan, China

**Keywords:** chronic disease count, community-dwelling older adults, fall timing, falls, recurrent falls

## Abstract

**Background:**

Chronic conditions and falls commonly co-occur in older populations. This study described fall occurrence, recurrent falls, and participant-level fall timing across chronic disease count groups in a single-city community sample.

**Methods:**

This cross-sectional survey included 610 community-dwelling adults aged ≥60 years in Neijiang, Sichuan, China. Self-reported falls during the preceding 12 months were classified by occurrence and frequency; recurrent falls were defined as ≥2 falls. One participant-level fall-timing category was analyzed per faller. Chronic disease count was an unweighted sum of seven disease domains. Crude and minimally adjusted logistic regression described count associations. Disease-specific crude associations, post-LASSO models, and an equal-duration morning-versus-afternoon comparison were exploratory analyses.

**Results:**

Overall, 303 participants (49.7%) reported falls and 96 (15.7%) reported recurrent falls. Fall occurrence was non-monotonic across count groups (52.9%, 42.2%, 47.8%, 56.6%, and 68.6% for 0, 1, 2, 3, and ≥4 diseases); corresponding recurrent-fall proportions were 9.6%, 12.9%, 16.5%, 19.7%, and 37.3%. The minimally adjusted OR for recurrent falls in the ≥4 group versus 0 was 4.85 (95% CI 2.06–11.46). The five-category timing distribution did not show clear evidence of an overall difference (Pearson *p* = 0.195). The morning/non-morning contrast had *p* = 0.024, and the equal-duration comparison included 203 fallers (108 morning and 95 afternoon; *p* = 0.011). Disease-specific estimates varied across diseases and outcomes. Post-LASSO findings remained exploratory.

**Conclusion:**

Fall-related characteristics differed across chronic disease count groups in this Neijiang sample, particularly with respect to recurrent falls. Timing differences depended on the comparison used. These cross-sectional findings are hypothesis-generating and do not establish temporal or causal relationships. Prospective, multicenter studies are required.

## 1 Introduction

Falls are a common public health problem among older adults (1), and their global incidence has increased as populations have aged (2). The World Health Organization estimates approximately 684,000 fatal falls and 37.3 million falls requiring medical attention worldwide each year (3). Falls are associated with subsequent limitations in activities of daily living (4). Describing fall history and recurrence in community samples can therefore help characterize the circumstances in which falls are reported.

Chronic non-communicable diseases are common in older adults and are associated with poorer function and quality of life (5, 6). The World Health Organization estimates that non-communicable diseases accounted for at least 43 million deaths in 2021 (7). Multimorbidity and polypharmacy frequently co-occur, although prevalence estimates depend substantially on the conditions and medications included in their definitions (8). Chronic disease count is an aggregate, unweighted measure of accumulated conditions. It does not characterize disease severity, and a multicenter study in Sri Lanka illustrates that aggregate counts and medication measures do not consistently show associations with reported falls across settings (9).

Longitudinal studies have reported associations between multimorbidity and falls, including bidirectional associations in a Chinese cohort (10). Balance-related mechanisms involve musculoskeletal, central nervous, and sensory systems (11). In a five-country prospective study, a higher baseline number of chronic diseases was associated with a higher rate of subsequent falls (12). Disease patterns have also shown differing associations with falls in Chinese community samples (13). These findings motivate examining an aggregate count alongside more specific fall-related characteristics, while recognizing that recurrence and time-of-day distributions describe different aspects of reported fall history.

This study examined fall occurrence, fall frequency, recurrent falls, and participant-level fall timing across chronic disease count groups in Neijiang. Descriptive comparisons and crude and minimally adjusted count associations were the main exposure analyses. Disease-specific crude associations, multidomain attenuation models, post-LASSO associations, and an equal-duration timing comparison were secondary exploratory analyses.

## 2 Methods

### 2.1 Study design, setting, and participants

This cross-sectional study was conducted among community-dwelling older adults in Neijiang, Sichuan Province, China, from March 2026 to May 2026. The study was reported in accordance with the Strengthening the Reporting of Observational Studies in Epidemiology guidelines. Data were collected using a structured questionnaire administered through Wenjuanxing, a China-based online survey platform accessible via web browsers and WeChat. Eligible participants were adults aged 60 years or older who lived in the community, were able to understand the questionnaire content, and voluntarily agreed to participate. Participants were excluded if they did not meet the age criterion, were not community-dwelling older adults, or failed predefined quality-control checks. These checks included implausible eligibility information, logically inconsistent responses, and obvious response-pattern abnormalities. For participants who had difficulty completing the questionnaire independently, trained investigators or community staff provided standardized assistance without influencing their responses.

Wenjuanxing and WeChat facilitated community distribution, survey completion, and data completeness. This practical recruitment approach was not a probability sample of all older residents, and participation could depend on health status and digital access.

All Wenjuanxing items required for the analysis were mandatory, and included questionnaires had no item-level missing values; no imputation was performed. No *a priori* sample-size or power calculation was performed. The analytic sample comprised all eligible completed questionnaires collected during the predefined study period. All participants provided informed consent. The study was approved by the Ethics Committee of Neijiang Health Vocational College (Approval No. LLW-202603-01).

### 2.2 Measurements and variables

Sociodemographic variables included sex, age group (60–65, 66–70, 71–75, and >75 years), marital status, education, living arrangement, monthly income, medical insurance, smoking, alcohol consumption, and physical activity. Chronic disease count was calculated as the unweighted number of reported physician-diagnosed chronic disease categories: hypertension, diabetes, osteoarticular disease/osteoporosis, heart disease, cerebrovascular disease, hyperlipidemia, and other physician-diagnosed chronic disease. Each domain contributed 0 or 1 point; the other-disease domain contributed at most 1 point. Heart disease and cerebrovascular disease were counted separately but are presented together in Table 1 for descriptive purposes. Osteoarticular disease and osteoporosis constituted one domain and were not double-counted. The count did not incorporate disease severity, duration, complications, treatment intensity, or disease-specific functional impact. Groups were 0, 1, 2, 3, and ≥4, with ≥4 denoting four or more counted disease categories (Supplementary Table 12). Multimorbidity was defined as two or more chronic diseases (5) (Supplementary Table 13). Medication variables included medication use, central nervous system depressant medication use, and number of medications; the count did not capture drug-specific dose or duration.

**Table 1:** Participant characteristics according to fall status.

Items	Fall group	Non-fall group	*P*
	*n* = 303	*n* = 307	
Sex			0.104
Male	105 (34.7%)	126 (41.0%)	
Female	198 (65.3%)	181 (59.0%)	
Age group			0.439
60–65	102 (33.7%)	112 (36.5%)	
66–70	32 (10.6%)	37 (12.1%)	
71–75	74 (24.4%)	80 (26.1%)	
>75	95 (31.4%)	78 (25.4%)	
Smoking			0.140
Yes	84 (27.7%)	102 (33.2%)	
No	219 (72.3%)	205 (66.8%)	
Alcohol consumption			0.182
Yes	96 (31.7%)	113 (36.8%)	
No	207 (68.3%)	194 (63.2%)	
Hypertension			0.492
Yes	121 (39.9%)	131 (42.7%)	
No	182 (60.1%)	176 (57.3%)	
Diabetes			0.028
Yes	82 (27.1%)	60 (19.5%)	
No	221 (72.9%)	247 (80.5%)	
Osteoarticular disease/osteoporosis			0.064
Yes	106 (35.0%)	86 (28.0%)	
No	197 (65.0%)	221 (72.0%)	
Cardiovascular and cerebrovascular diseases			0.125
Yes	77 (25.4%)	62 (20.2%)	
No	226 (74.6%)	245 (79.8%)	
Hyperlipidemia			0.225
Yes	30 (9.9%)	40 (13.0%)	
No	273 (90.1%)	267 (87.0%)	
Other physician-diagnosed chronic disease			0.133
Yes	40 (13.2%)	54 (17.6%)	
No	263 (86.8%)	253 (82.4%)	
CNS depressant medications			0.006
Yes	21 (6.9%)	7 (2.3%)	
No	282 (93.1%)	300 (97.7%)	
Number of medications			<0.001
0	86 (28.4%)	87 (28.3%)	
1	121 (39.9%)	153 (49.8%)	
2	62 (20.5%)	59 (19.2%)	
≥3	34 (11.2%)	8 (2.6%)	
GAD-7 score	1 (0–7)	1 (0–3)	0.003
PHQ-9 score	4 (1–9)	4 (0–8)	0.045

Values are *n* (%) or median (IQR). Categorical *p*-values are Pearson chi-square tests without continuity correction; score comparisons use Mann–Whitney *U* tests. CNS, central nervous system; GAD-7, Generalized Anxiety Disorder-7; PHQ-9, Patient Health Questionnaire-9. Heart disease and cerebrovascular disease were counted separately in the chronic disease count but are presented together in table for descriptive purposes. Osteoarticular disease/osteoporosis contributes at most one point, as does other physician-diagnosed chronic disease. The oldest age group is >75 years. Logistic Wald *p*-values in Supplementary Table 11 can differ slightly from these group-comparison *p*-values.

Functional and sensory characteristics were assessed by structured self-report items: frailty, lower limb weakness, balance ability, visual decline, hearing decline, dizziness, sleep insufficiency, attention impairment, nighttime toileting, and assistance with daily activities. Frailty was self-rated as none, mild, or severe, and balance as good, fair, or poor. Environmental and care-related items included footwear and bed-height suitability, surrounding environment, walking aids, intravenous infusion access, room lighting, community familiarity, door thresholds, slippery bathroom floors, and community handrails. Physical activity was a binary item. Psychological symptoms were assessed using GAD-7 and PHQ-9 (14, 15), with each item scored 0–3 and higher totals indicating more symptoms. For both scales, scores of 0–4 indicated none/minimal symptoms, 5–9 mild symptoms, and ≥10 moderate-to-severe symptoms; the latter pooled the moderate and higher severity categories. Binary anxiety symptoms and depressive symptoms were defined by GAD-7 ≥ 5 and PHQ-9 ≥ 5, respectively. These thresholds described symptoms and did not establish clinical diagnoses.

### 2.3 Fall-related outcomes

Fall-related outcomes were assessed based on self-reported fall history within the past year. A fall was defined as an event in which a participant unintentionally came to rest on the ground, floor, or a lower level. Participants were first classified as fallers or non-fallers according to whether they had experienced at least one fall within the past year. Fall frequency was recorded as the number of fall events within the past year and was categorized as 0, 1, 2, and 3 or more falls. Recurrent falls were defined as two or more fall events within the past year. Accordingly, participants were classified as having recurrent falls or no recurrent falls.

Among the 303 fallers, one participant-level timing category was available: 00:00–<06:00, 06:00–<12:00, 12:00–<18:00, 18:00–<22:00, or 22:00–<24:00. Each lower boundary was included and each upper boundary excluded, so exactly 06:00 belonged to morning and exactly 12:00 to afternoon. For participants with recurrent falls, one participant-level fall-timing category was available for analysis; event-specific timing across multiple fall events was not available. The five-category distribution was the principal timing description. The secondary morning/non-morning contrast compared 06:00–<12:00 (6 h) with the remaining 18 h. These boundaries represented conventional daily periods and were not selected to optimize the observed *p*-values. Similar intervals have been used in older-adult fall research: Kim et al. (16) used 06:00–12:00 and 12:00–18:00, Sheridan et al. (17) used 06:00–11:59 and 12:00–17:59, and Choi et al. (18) defined morning as 06:01–12:00 in an older-adult hip-fracture study. These conventions support the timing labels but do not establish biological thresholds or a validated clinical subtype.

### 2.4 Statistical analysis

Continuous variables were summarized as medians and interquartile ranges, and categorical variables as counts and percentages. Categorical comparisons in Tables 1, 2, 3 and Supplementary Table 1 were performed using Pearson chi-square tests without continuity correction. Mann–Whitney *U* tests compared GAD-7 and PHQ-9 score distributions. Logistic regression results were reported as odds ratios and Wald 95% confidence intervals, using exp.(β ± 1.96 SE). Crude count-group and multimorbidity odds ratios were calculated from the corresponding 2 × 2 contingency tables. Model 1 adjusted for sex, age group, smoking, alcohol consumption, and physical activity. The reference was 0 diseases for fall occurrence and recurrent falls. Table 4 pooled 0–1 diseases to increase reference information in the faller-only analysis; this pooling did not remove all sparse-cell concerns. Supplementary Table 4 and the post-LASSO timing model used 0 as reference. Ordered-category trend tests used scores 0, 1, 2, 3, and 4 for the five count groups, and 0, 1, 2, and 3 for the pooled-reference groups in Table 4. These tests did not establish a linear biological dose–response.

**Table 2:** Fall occurrence and recurrent falls by chronic disease count.

Items	0 diseases	1 disease	2 diseases	3 diseases	≥4 diseases	*P*
	*n* = 136	*n* = 232	*n* = 115	*n* = 76	*n* = 51	
Fall occurrence						0.006
No	64 (47.1%)	134 (57.8%)	60 (52.2%)	33 (43.4%)	16 (31.4%)	
Yes	72 (52.9%)	98 (42.2%)	55 (47.8%)	43 (56.6%)	35 (68.6%)	
Fall frequency						<0.001
0	64 (47.1%)	134 (57.8%)	60 (52.2%)	33 (43.4%)	16 (31.4%)	
1	59 (43.4%)	68 (29.3%)	36 (31.3%)	28 (36.8%)	16 (31.4%)	
2	6 (4.4%)	25 (10.8%)	14 (12.2%)	11 (14.5%)	12 (23.5%)	
≥3	7 (5.1%)	5 (2.2%)	5 (4.3%)	4 (5.3%)	7 (13.7%)	
Recurrent falls						<0.001
No	123 (90.4%)	202 (87.1%)	96 (83.5%)	61 (80.3%)	32 (62.7%)	
Yes	13 (9.6%)	30 (12.9%)	19 (16.5%)	15 (19.7%)	19 (37.3%)	

Values are *n* (%), with all participants in the count group as denominator. Recurrent falls were ≥2 falls during the preceding year; the comparison group includes both non-fallers and single fallers. *p*-values were calculated using Pearson chi-square tests without continuity correction. The frequency comparison had two of 20 expected cells below 5 and none below 1.

**Table 3:** Fall timing by chronic disease count among fallers.

Fall timing	0 diseases	1 disease	2 diseases	3 diseases	≥4 diseases	*P*
	n = 72	n = 98	n = 55	n = 43	n = 35	
Five-category timing						0.195
00:00–<06:00	11 (15.3%)	9 (9.2%)	5 (9.1%)	6 (14.0%)	3 (8.6%)	
06:00–<12:00	35 (48.6%)	38 (38.8%)	14 (25.5%)	13 (30.2%)	8 (22.9%)	
12:00–<18:00	14 (19.4%)	32 (32.7%)	20 (36.4%)	12 (27.9%)	17 (48.6%)	
18:00–<22:00	9 (12.5%)	16 (16.3%)	12 (21.8%)	9 (20.9%)	5 (14.3%)	
22:00–<24:00	3 (4.2%)	3 (3.1%)	4 (7.3%)	3 (7.0%)	2 (5.7%)	
Morning vs non-morning timing						0.024
Morning fall, 06:00–<12:00	35 (48.6%)	38 (38.8%)	14 (25.5%)	13 (30.2%)	8 (22.9%)	
Non-morning fall	37 (51.4%)	60 (61.2%)	41 (74.5%)	30 (69.8%)	27 (77.1%)	

Values are *n* (%) of fallers within each count group. The five-category distribution is the primary timing description. *p*-values were calculated using Pearson chi-square tests. For the sparse five-category table, a conditional Monte Carlo Pearson test with fixed row and column margins gave *p* = 0.196 (200,000 replicates; seed 20,260,909). Morning/non-morning is a secondary 6-h versus 18-h contrast; no activity-exposure denominator was available. See Supplementary Tables 9, 10 for the equal-duration sensitivity analysis and summary of timing comparisons.

**Table 4:** Association between chronic disease count and non-morning falls among fallers.

Chronic disease count	Crude OR (95% CI)	Model 1 OR (95% CI)
0–1 chronic disease	Reference	Reference
2 chronic diseases	2.20 (1.12–4.34)	1.56 (0.74–3.29)
3 chronic diseases	1.74 (0.85–3.56)	1.25 (0.56–2.75)
≥4 chronic diseases	2.54 (1.09–5.92)	1.90 (0.76–4.77)
*P* for trend	0.008	0.156

The outcome is non-morning versus morning timing among 303 fallers. The 0–1 reference contains 170 fallers (73 morning, 97 non-morning), compared with 72 fallers (35 morning, 37 non-morning) in the unpooled 0 group. Crude ORs and Wald CIs were calculated from the contingency counts in Table 3. Model 1 adjusts for sex, age group, smoking, alcohol consumption, and physical activity. The pooled groups use ordinal scores 0–3 for *P* for trend. The exploratory multidomain attenuation model is presented in Supplementary Table 6.

Model 2 was an exploratory multidomain attenuation model (Supplementary Table 6). Its purpose was to describe changes in count associations after adding medication-related, functional, sensory, psychological, and environmental variables to Model 1. Some added variables could be proximal factors or potential mediators. This model was not a formal mediation analysis or a standard confounder-adjusted total-effect model (19). Changes in logistic ORs can also reflect non-collapsibility (20). The variable-category specification implied approximately 50 nominal slope parameters for recurrent falls and approximately 1.9 recurrent-fall events per nominal parameter (96/50). The pooled-reference timing model implied approximately 49 nominal parameters, with 195/49 = 3.98 non-morning participants and 108/49 = 2.20 morning comparators per parameter. These design-based counts excluded the intercept and did not represent fitted design-matrix ranks (Supplementary Tables 7, 8).

Disease-specific exploratory crude associations were summarized separately (Supplementary Table 11). Fall-occurrence ORs and Wald CIs were calculated from Table 1 for six descriptive disease categories. Disease-specific crude associations with recurrent falls and non-morning timing were summarized for hypertension, diabetes, osteoarticular disease/osteoporosis, and the combined cardiovascular/cerebrovascular category. Each comparison was Yes versus No; occurrence and recurrence used all participants, while timing used fallers only. These analyses did not adjust for covariates or separate heart disease from cerebrovascular disease within the combined descriptive indicator.

Variables with univariable *p* < 0.05 were considered for logistic LASSO in R 4.5.1 using glmnet, a binomial family, dummy coding, standardized inputs, cross-validation, and selection at *λ*_1se_. The selected variables were subsequently fitted by logistic regression with their full factor categories. Selection and estimation used the same dataset, so conventional ORs, CIs, and *p*-values did not account for selection uncertainty and were not confirmatory post-selection inference (21). No independent validation or bootstrap selection-stability analysis was performed (Supplementary Table 14).

The equal-duration sensitivity analysis compared 06:00–<12:00 with 12:00–<18:00 across count groups using a 2 × 5 Pearson test. All expected counts exceeded 5. For the sparse five-category table, a fixed-margin conditional Monte Carlo Pearson test used 200,000 random tables and seed 20,260,909. Monte Carlo and contingency-table analyses were implemented in Python 3.12 using SciPy 1.16.2; grouped-binomial trend analyses were conducted in R. No formal adjustment for multiple comparisons was applied because the analyses were exploratory. Accordingly, secondary and borderline findings were interpreted cautiously and should be considered hypothesis-generating.

## 3 Results

### 3.1 Baseline characteristics of participants

A total of 657 questionnaires were initially collected. After excluding participants who did not meet the age criterion (*n* = 9), were not community-dwelling older adults (*n* = 4), or failed quality-control checks (*n* = 34), 610 eligible participants were included in the final analysis. Among them, 303 participants reported at least one fall within the past year, whereas 307 participants reported no falls. The participant flow diagram and analytic groups are shown in Figure 1.

**Figure 1:**
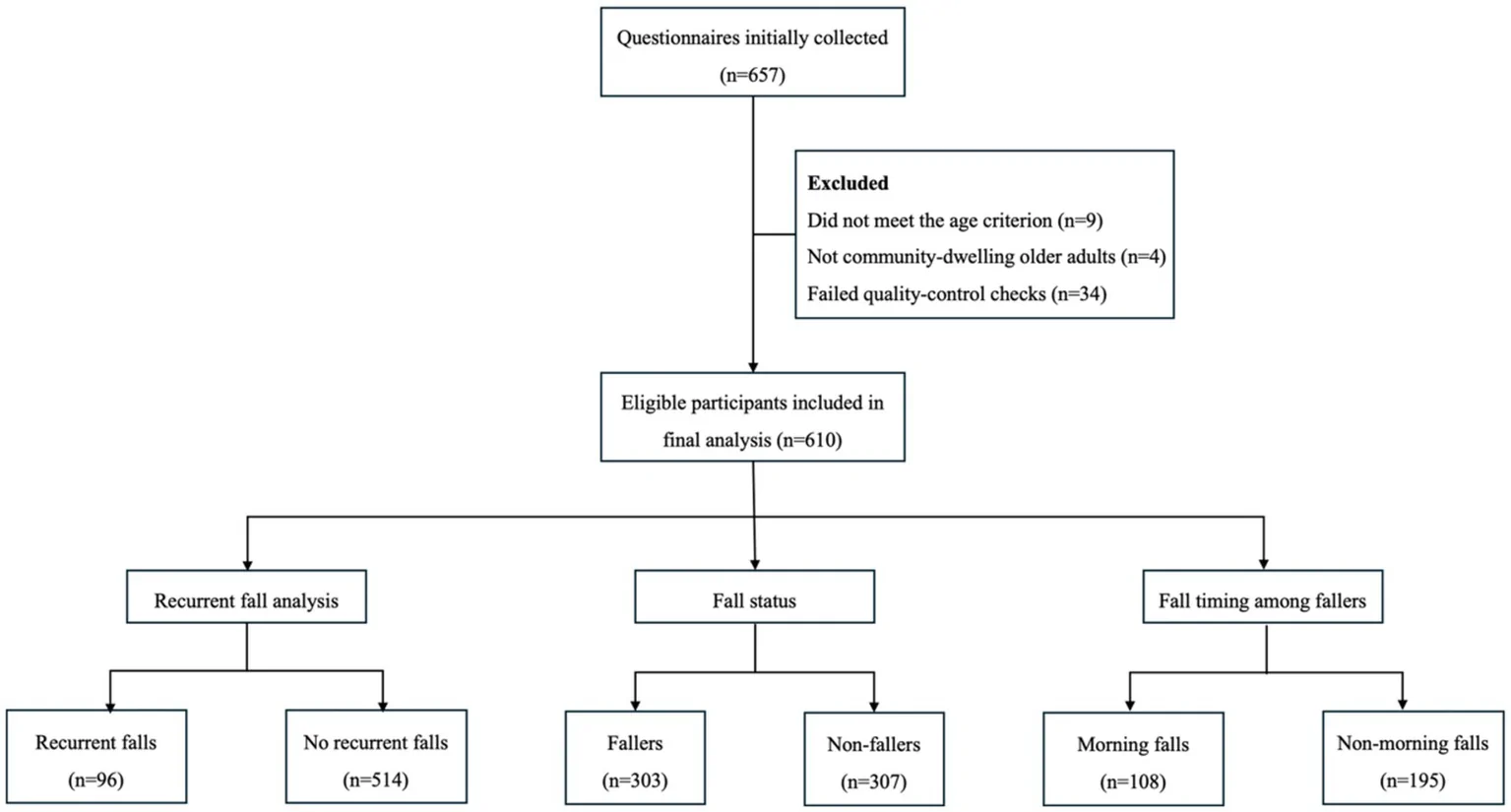
Participant flow diagram and analytic groups. Flow diagram showing questionnaire screening, final inclusion, fall status classification, recurrent fall classification, and fall timing classification among fallers.

Among 610 participants, 231 were men (37.9%) and 379 women (62.1%); 214 (35.1%) were aged 60–65 years. Table 1 describes the sample by fall status. Diabetes was reported by 27.1% of fallers and 19.5% of non-fallers, and central nervous system depressant medication use by 6.9 and 2.3%, respectively. Other disease-category proportions showed less clear between-group differences. The *p* value for hyperlipidemia was 0.225. GAD-7 and PHQ-9 score distributions and the corresponding rank-test *p*-values are presented. Additional functional, environmental, and care-related characteristics appear in Supplementary Table 1.

### 3.2 Chronic disease count and fall-related outcomes

#### 3.2.1 Fall occurrence and recurrent falls according to chronic disease count

Fall occurrence was 52.9%, 42.2%, 47.8%, 56.6%, and 68.6% across the 0, 1, 2, 3, and ≥4 disease groups, respectively (Table 2 and Figure 2). The overall comparison had *p* = 0.006, but occurrence did not follow a strictly monotonic pattern. Fall frequency and recurrent falls also differed across groups (both *p* < 0.001). Recurrent-fall proportions were 9.6%, 12.9%, 16.5%, 19.7%, and 37.3%, constituting a clearer graded descriptive pattern without establishing causality.

**Figure 2:**
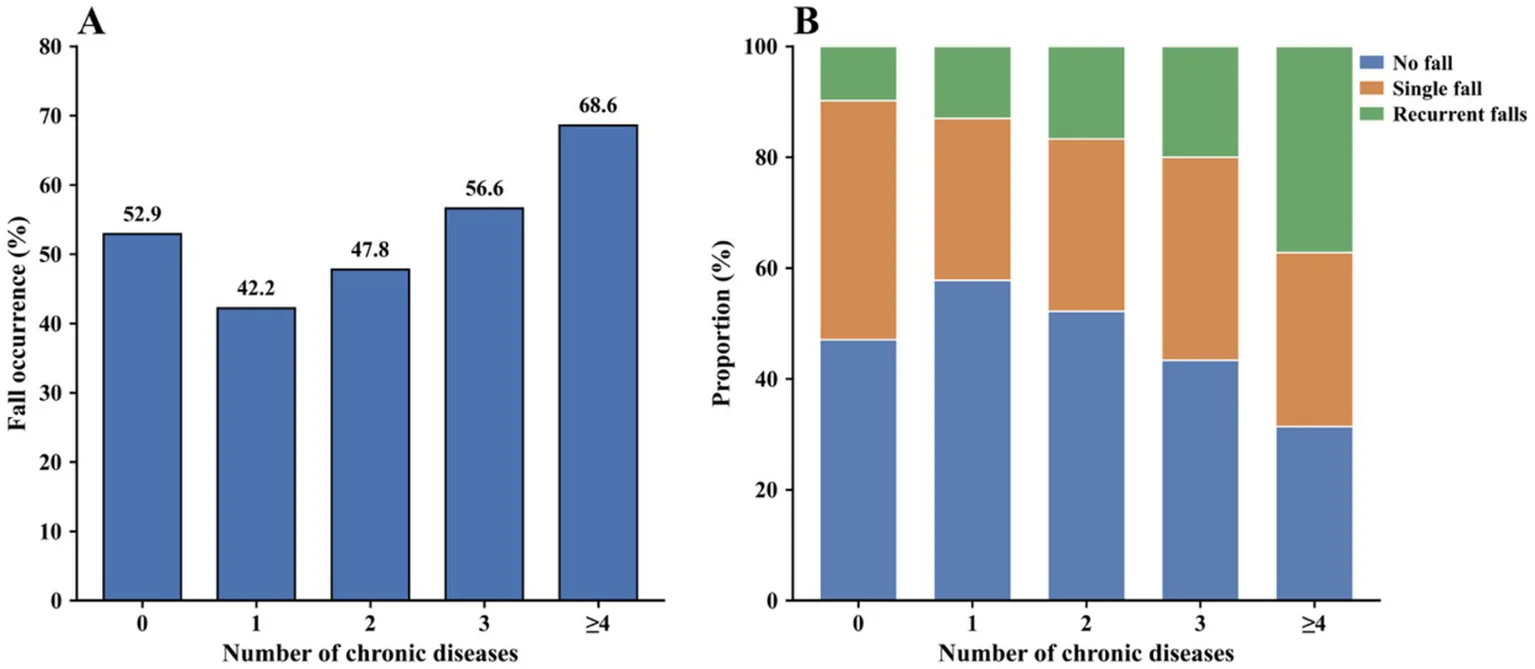
Fall occurrence and recurrent falls by chronic disease count. **(A)** Proportion reporting ≥1 fall in the preceding year by recorded chronic disease count. **(B)** Distribution of no fall, single fall, and recurrent falls. These are participant proportions, not incidence rates.

#### 3.2.2 Associations of chronic disease count with fall occurrence and recurrent falls

In Table 5, the crude OR for fall occurrence in the ≥4 group versus 0 was 1.94 (95% CI 0.98–3.84; *P* ≈ 0.056). The Model 1 estimate was 1.85 (0.90–3.84). The estimate was higher but imprecise and did not meet the conventional 0.05 threshold. The lower occurrence in the 1-disease group and higher occurrence in the ≥4 group do not constitute a stable dose–response pattern.

**Table 5:** Association between chronic disease count and fall occurrence.

Chronic disease count	Crude OR (95% CI)	Model 1 OR (95% CI)
0 chronic diseases	Reference	Reference
1 chronic disease	0.65 (0.42–1.00)	0.61 (0.39–0.96)
2 chronic diseases	0.81 (0.50–1.34)	0.92 (0.54–1.56)
3 chronic diseases	1.16 (0.66–2.04)	1.23 (0.68–2.25)
≥4 chronic diseases	1.94 (0.98–3.84)	1.85 (0.90–3.84)
*P* for trend	0.027	0.018

Crude ORs and Wald 95% CIs were calculated from the contingency counts in Table 2. Model 1 adjusts for sex, age group, smoking, alcohol consumption, and physical activity. *P* for trend is an ordered-category test using scores 0–4. Model 2 is presented in Supplementary Table 6 as an exploratory multidomain attenuation model.

For recurrent falls, the crude OR in the ≥4 group versus 0 was 5.62 (95% CI 2.51–12.57), and the Model 1 estimate was 4.85 (2.06–11.46). Corresponding estimates for the 3-disease group were 2.33 (1.04–5.20) and 2.18 (0.94–5.06) (Table 6). The recorded group pattern was more pronounced for recurrent falls than for occurrence, but it cannot be interpreted as a causal dose–response. The expanded attenuation estimates in Supplementary Table 6 do not establish an independent effect or absence of association.

**Table 6:** Association between chronic disease count and recurrent falls.

Chronic disease count	Crude OR (95% CI)	Model 1 OR (95% CI)
0 chronic diseases	Reference	Reference
1 chronic disease	1.41 (0.71–2.80)	1.27 (0.63–2.59)
2 chronic diseases	1.87 (0.88–3.98)	1.93 (0.87–4.25)
3 chronic diseases	2.33 (1.04–5.20)	2.18 (0.94–5.06)
≥4 chronic diseases	5.62 (2.51–12.57)	4.85 (2.06–11.46)
*P* for trend	<0.001	<0.001

Recurrent falls were ≥2 falls versus 0–1 falls among all 610 participants. Crude ORs and Wald 95% CIs were calculated from the contingency counts in Table 2. Model 1 adjusts for sex, age group, smoking, alcohol consumption, and physical activity. *P* for trend uses ordinal scores 0–4. The exploratory multidomain attenuation model is presented in Supplementary Table 6.

### 3.3 Fall timing according to chronic disease count among fallers

#### 3.3.1 Fall timing distribution according to chronic disease count

The five recorded timing categories accounted for 34, 108, 95, 51, and 15 fallers, respectively (Table 3). The five-category fall-timing distribution did not show clear evidence of an overall difference across chronic disease count groups (Pearson *χ*^2^ = 20.582, 16 df, *p* = 0.195). Because seven of 25 expected counts were below 5, a fixed-margin conditional Monte Carlo Pearson test was also performed (*p* = 0.196). These categories describe participants, rather than every fall event.

The exploratory morning/non-morning comparison had *p* = 0.024. Morning proportions across the five count groups were 48.6%, 38.8%, 25.5%, 30.2%, and 22.9%, showing a non-monotonic pattern (Figure 3). The equal-duration morning-versus-afternoon comparison included 203 fallers, with counts of 35/14, 38/32, 14/20, 13/12, and 8/17 across count groups. Pearson *χ*^2^(4) = 13.07, *p* = 0.011; the remaining 100 fallers were outside these windows. Differences were therefore also observed within the restricted comparison of two equal-length periods, but this sensitivity result did not establish an overall five-category difference or validate the broader morning/non-morning contrast.

**Figure 3:**
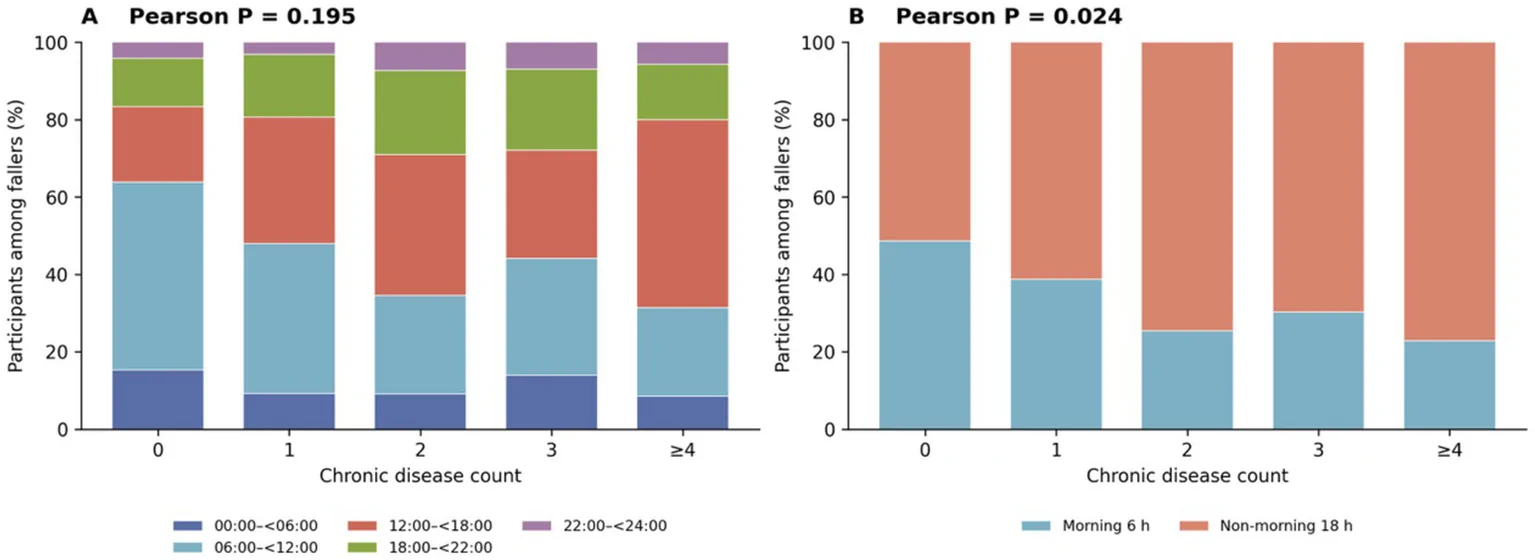
Fall timing by chronic disease count among fallers. **(A)** Five-category participant-level timing distribution; Pearson *p* = 0.195. **(B)** Exploratory morning/non-morning distribution; Pearson *p* = 0.024. Bars use the Table 3 counts. The binary windows cover 6 and 18 h, respectively. Neither panel estimates an hourly fall rate.

#### 3.3.2 Association of chronic disease count with non-morning falls among fallers

Among fallers, the analysis in Table 4 used the 0–1 disease group as the pooled reference. Crude ORs for non-morning versus morning timing were 2.20 (95% CI 1.12–4.34), 1.74 (0.85–3.56), and 2.54 (1.09–5.92) for the 2, 3, and ≥4 groups, respectively. The Model 1 estimates were 1.56 (0.74–3.29), 1.25 (0.56–2.75), and 1.90 (0.76–4.77). These imprecise minimally adjusted estimates do not provide a stable graded pattern. Expanded attenuation results are presented separately in Supplementary Table 6.

### 3.4 Exploratory post-LASSO associations with recurrent falls

Recurrent falls were reported by 96 of 610 participants (15.7%); 514 reported zero or one fall. Univariable associations are presented in Supplementary Table 2, including the multimorbidity estimate of 2.12 (95% CI 1.36–3.29; *p* < 0.001). The LASSO analysis considered 35 candidate variables, represented by 58 nominal dummy-coded features, with *λ*_min_ = 0.006997 and *λ*_1se_ = 0.013102. At *λ*_1se_, 16 nonzero features mapped to 15 variables, which were then fitted using their full factor categories. Figures 4A,B show the coefficient paths and cross-validation curve. The complete post-LASSO model contains 26 nominal slope coefficients, giving 96/26 = 3.69 recurrent-fall events per nominal parameter (Supplementary Table 7).

**Figure 4:**
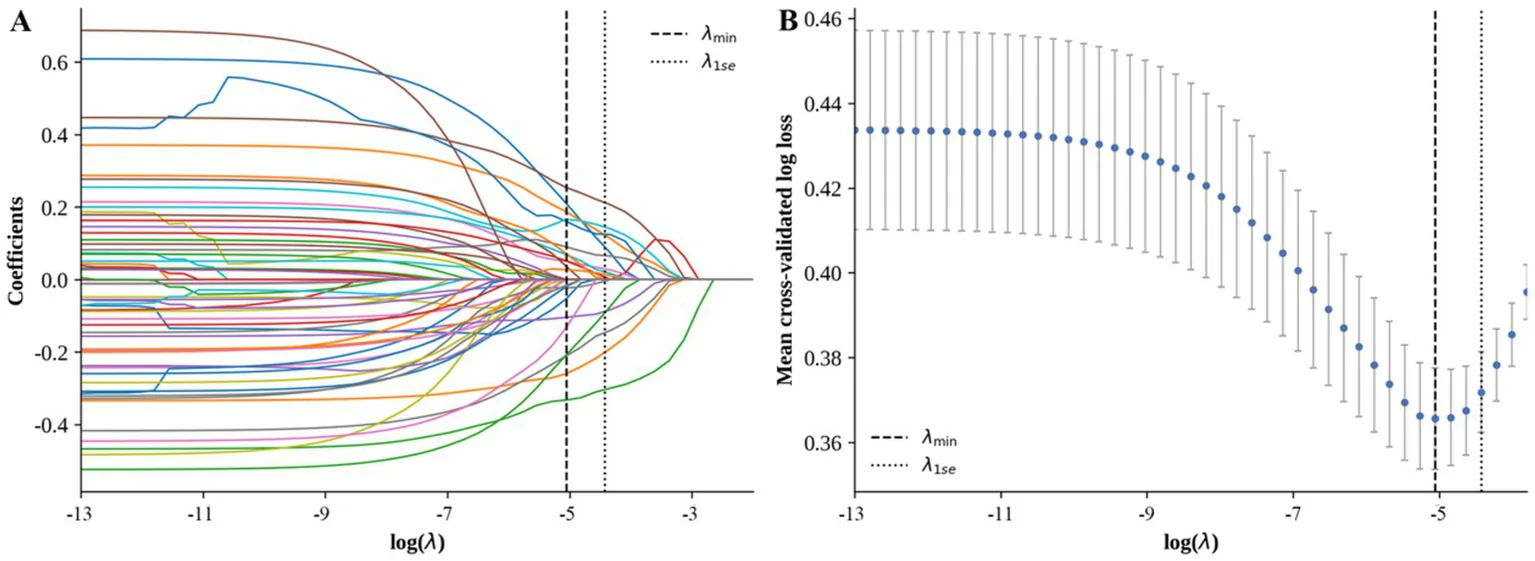
LASSO variable selection for recurrent falls. **(A)** Coefficient paths. **(B)** Cross-validation curve. Both horizontal axes are log(λ); *λ*_min_ = 0.006997 and *λ*_1se_ = 0.013102 are reported on the untransformed *λ* scale. Dashed and dotted lines indicate *λ*_min_ and *λ*_1se_, respectively. Selection was followed by exploratory association analysis in the same dataset.

The post-LASSO model identified several variables showing exploratory associations with recurrent falls (Table 7; complete model in Supplementary Table 3). Estimates included frequent dizziness (OR 4.09, 95% CI 1.62–10.32), mild frailty (OR 2.61, 95% CI 1.24–5.49), severe frailty (OR 3.41, 95% CI 1.23–9.44), anxiety symptoms (OR 1.41, 95% CI 1.04–1.90), no versus yes lower limb weakness (OR 0.47, 95% CI 0.23–0.94), and no versus yes slippery bathroom floor (OR 0.58, 95% CI 0.34–0.98). Income estimates were 0.62 (0.32–1.18), 0.73 (0.34–1.60), and 0.25 (0.09–0.67) for successively higher categories relative to <2000 CNY, without a monotonic gradient.

**Table 7:** Selected rows from the exploratory post-LASSO recurrent-fall association model.

Items	OR (95% CI)	*p*-value
Use of walking aid		
Yes	Reference	
No	0.36 (0.19–0.69)	0.002
Dizziness		
None	Reference	
Occasional	1.46 (0.72–2.99)	0.294
Frequent	4.09 (1.62–10.32)	0.003
Lower limb weakness		
Yes	Reference	
No	0.47 (0.23–0.94)	0.033
Slippery bathroom floor		
Yes	Reference	
No	0.58 (0.34–0.98)	0.041
Anxiety symptoms		
No	Reference	
Yes	1.41 (1.04–1.90)	0.025
Frailty		
None	Reference	
Mild	2.61 (1.24–5.49)	0.012
Severe	3.41 (1.23–9.44)	0.018
Monthly income		
<2000 CNY	Reference	
2000–4,000 CNY	0.62 (0.32–1.18)	0.146
4,000–6,000 CNY	0.73 (0.34–1.60)	0.438
≥6,000 CNY	0.25 (0.09–0.67)	0.006

Recurrent falls were ≥2 falls versus 0–1 falls. All 15 variables and all levels are provided in Supplementary Table 3. Because variable selection and subsequent logistic regression were performed in the same dataset, the ORs, 95% CIs, and *p*-values are exploratory post-selection association estimates and should not be interpreted as confirmatory inference.

### 3.5 Exploratory post-LASSO associations with non-morning timing

Among the 303 fallers, 108 reported morning timing and 195 non-morning timing. Supplementary Table 4 presents univariable associations, including the multimorbidity OR of 2.11 (95% CI 1.29–3.44; *p* = 0.003). The LASSO analysis considered 34 candidate variables. After dummy coding of categorical variables, LASSO regression was performed on the resulting design matrix. *λ*_min_ was 0.007024 and *λ*_1se_ was 0.011655; 10 selected features mapped to 10 variables (Figure 5). Refitting full factor categories produced 21 nominal slope coefficients, corresponding to 195/21 = 9.29 non-morning participants per parameter and 108/21 = 5.14 in the smaller morning group (Supplementary Table 7).

**Figure 5:**
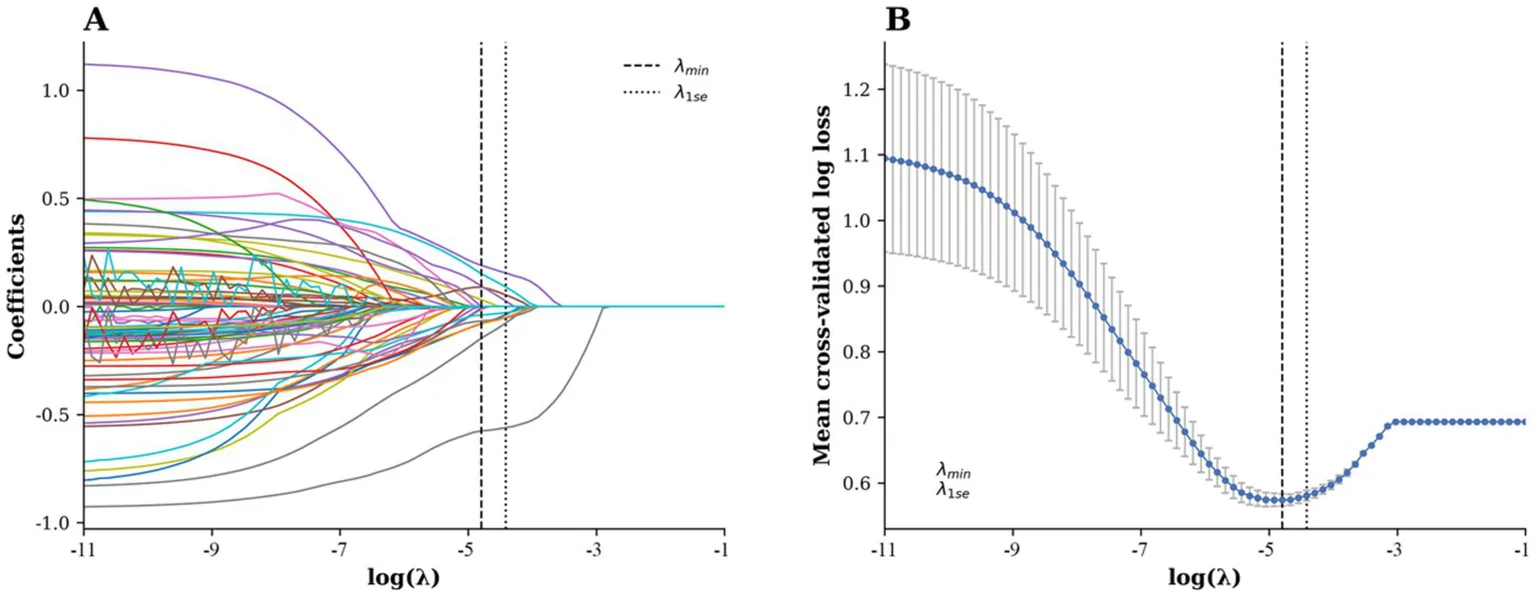
LASSO variable selection for non-morning falls among fallers. **(A)** Coefficient paths. **(B)** Cross-validation curve for the faller-only timing analysis. Both horizontal axes are log(λ); *λ*_min_ = 0.007024 and *λ*_1se_ = 0.011655 are on the untransformed *λ* scale. Dashed and dotted lines indicate *λ*_min_ and *λ*_1se_, respectively.

Table 8 and Supplementary Table 5 show the complete exploratory post-LASSO timing model, including levels whose CIs cross 1. ORs for two and ≥3 medications were 2.18 (95% CI 1.08–4.42) and 2.76 (1.13–6.72); for fair and poor balance, 2.05 (1.12–3.77) and 3.48 (1.31–9.25); and for mild and severe hearing decline, 3.96 (2.08–7.55) and 5.72 (2.18–15.01). The direction of the physical-activity estimate differed between the univariable analysis (Yes versus No: OR 0.46, 95% CI 0.29–0.75; *p* = 0.002) and the post-LASSO multivariable analysis (OR 1.88, 1.08–3.27; *p* = 0.025).

**Table 8:** Exploratory post-LASSO associations with non-morning timing among fallers.

Items	OR (95% CI)	*P*
Chronic disease count		
0	Reference	
1	1.08 (0.52–2.23)	0.840
2	1.31 (0.52–3.29)	0.563
3	1.42 (0.51–3.95)	0.501
≥4	1.56 (0.47–5.19)	0.469
Number of medications		
0	Reference	
1	1.34 (0.72–2.49)	0.356
2	2.18 (1.08–4.42)	0.030
≥3	2.76 (1.13–6.72)	0.026
Frailty		
None	Reference	
Mild	1.29 (0.68–2.45)	0.435
Severe	1.61 (0.55–4.72)	0.383
Balance ability		
Good	Reference	
Fair	2.05 (1.12–3.77)	0.020
Poor	3.48 (1.31–9.25)	0.012
Dizziness		
None	Reference	
Occasional	1.76 (1.01–3.07)	0.046
Frequent	2.89 (1.12–7.46)	0.028
Hearing decline		
None	Reference	
Mild	3.96 (2.08–7.55)	<0.001
Severe	5.72 (2.18–15.01)	<0.001
Attention impairment		
None	Reference	
Occasional	1.48 (0.78–2.81)	0.230
Frequent	1.62 (0.58–4.51)	0.356
Physical activity		
No	Reference	
Yes	1.88 (1.08–3.27)	0.025
Familiarity with community environment		
Yes	Reference	
No	1.54 (0.79–3.02)	0.208
Community handrail availability		
Sufficient	Reference	
Basic	1.83 (1.02–3.30)	0.043
Insufficient	2.41 (1.19–4.89)	0.015

Non-morning versus morning timing is evaluated only among fallers. The chronic disease count reference is 0, unlike the 0–1 reference in Table 4. Because variable selection and subsequent logistic regression were performed in the same dataset, the ORs, 95% CIs, and *p*-values are exploratory post-selection association estimates and should not be interpreted as confirmatory inference. The complete model is also provided in Supplementary Table 5; the physical-activity direction differs from the univariable estimate in Supplementary Table 4.

### 3.6 Exploratory disease-specific associations

Supplementary Table 11 presents 14 crude disease-outcome estimates. For fall occurrence, diabetes had OR 1.53 (95% CI 1.05–2.23), while the other five intervals included 1. For recurrent falls, estimates were 1.60 (1.03–2.47) for hypertension, 1.54 (0.95–2.49) for diabetes, 1.99 (1.27–3.10) for osteoarticular disease/osteoporosis, and 2.39 (1.50–3.82) for the combined cardiovascular/cerebrovascular category. Corresponding non-morning estimates were 1.65 (1.01–2.69), 1.89 (1.07–3.32), 1.90 (1.14–3.19), and 1.42 (0.81–2.47). These exploratory crude comparisons showed differing patterns across diseases and outcomes; they did not establish independent disease effects.

## 4 Discussion

In this cross-sectional Neijiang sample, recurrent-fall proportions showed clearer group differences than fall occurrence, which was not strictly monotonic. Timing findings depended on the comparison: the five-category distribution did not show clear overall evidence of heterogeneity, whereas the morning/non-morning and equal-duration comparisons showed differences within their respective contrasts. The disease-specific estimates varied across diseases and outcomes, and the post-LASSO associations were exploratory. None of these findings establishes a causal sequence.

Prospective studies have reported associations of multimorbidity with fall rates (12) and of recurrent falls with physical, functional, and psychological health (22). The present recurrent-fall pattern is compatible with accumulated conditions co-occurring with persistent vulnerability, but the lower occurrence in the 1-disease group than in the 0 group argues against interpreting all outcomes as a uniform dose–response. Sampling variation, disease composition, measurement error, and residual confounding remain possible explanations.

The exploratory multidomain attenuation model described how count estimates changed after inclusion of more proximal domains. Functional status, medications, and psychological measures could be consequences of chronic conditions or prior falls, and controlling them could overadjust an association (19). Differences between logistic ORs can also reflect non-collapsibility (20). With approximately 50 nominal parameters and only 96 recurrent-fall events, overparameterization further limits interpretation. Attenuation does not quantify mediation, establish an independent effect, or negate the crude and minimally adjusted patterns.

The disease-specific crude results complemented the aggregate count. Diabetes showed higher fall-occurrence odds, while hypertension, osteoarticular disease/osteoporosis, and the combined cardiovascular/cerebrovascular category showed higher recurrent-fall estimates. Non-morning estimates followed another pattern. These differences are consistent with disease heterogeneity but were not formal between-disease coefficient tests. Differences in statistical significance do not themselves demonstrate different effect sizes. The crude analyses remain susceptible to confounding and cannot identify a principal causal disease; the combined cardiovascular/cerebrovascular indicator also cannot isolate its components.

The recurrent-fall post-LASSO associations involved walking aids, dizziness, lower limb weakness, frailty, slippery bathroom floors, anxiety symptoms, and income. Previous reviews and longitudinal analyses describe falls as multifactorial (23–25), while home-hazard intervention trials provide separate evidence about prevention (26, 27). The walking-aid association should be interpreted as a non-causal correlate rather than evidence of a harmful effect of walking-aid use. GAD-7 anxiety symptoms are also distinct from concern about falling, the construct examined by Ellmers et al. (28). The inverse estimate observed only in the highest income category was not accompanied by a monotonic gradient across income levels. Although socioeconomic conditions have been associated with falls elsewhere (29), the present pattern may reflect residual confounding, sampling variability, or imprecision and remains exploratory.

The timing categories describe the context of one report per participant. Earlier studies used similar morning/afternoon periods in emergency-service fall records (17), community falls (16), and older-adult hip fractures (18). The equal-duration sensitivity result supported persistence of a difference when analysis was restricted to two 6-h windows, but it did not test the identical population or contrast as morning versus all non-morning periods. It excluded 100 fallers and did not account for time spent active in each window. It therefore cannot establish a validated timing subtype or event-specific timing patterns across recurrent falls.

The timing model described associations with medication count, balance, dizziness, hearing decline, activity, and community handrails. Prior research supports examining fall-risk-increasing drug classes (30), balance and dizziness (31), and hearing loss (32), without establishing mechanisms for this survey’s timing contrast. Notably, the direction of the physical-activity estimate differed between the univariable and post-LASSO multivariable analyses. This reversal may reflect confounding, suppression by correlated functional or environmental variables, or instability of the post-selection model. Physical activity was assessed using a crude binary measure without intensity, duration, location, activity type, or exposure time, unlike more detailed measures used elsewhere (33). Activity could also have changed after a fall. This finding should not be interpreted as evidence that physical activity itself increases fall risk.

These observations motivate prospective study of recurrence and event-level timing, rather than changes to clinical practice based on these models. Exercise and multifactorial prevention already have support from independent trials and guidelines (34, 35). These established recommendations provide clinical context but were not evaluated as interventions in the present survey. Reviews of fall-risk assessment show variable performance across measures and settings (36, 37); we did not evaluate whether adding timing or disease count improves clinical assessment. Associations involving income, housing, or community handrails are especially likely to depend on local social conditions and infrastructure.

The cross-sectional design cannot establish temporality or causality, and reverse causation is possible. Falls and timing were self-reported over 12 months, with potential recall error for non-injurious and recurrent events. Chronic diseases and functional, sensory, and environmental measures were also self-reported. Timing was participant-level; event-specific timing across recurrent falls was unavailable. Morning covered 6 h and non-morning 18 h, with heterogeneous nighttime, afternoon, and evening contexts. Even the equal-duration comparison lacked activity-time denominators. The unweighted count omitted disease severity, duration, and complications; medication and activity measurements were coarse.

The multidomain attenuation model included possible mediators and approximately 50 nominal parameters for recurrent falls, giving approximately 1.9 events per nominal parameter. Overadjustment and overparameterization therefore limit its stability; nominal dimensions do not establish effective fitted ranks. Same-sample univariable screening, LASSO selection, and subsequent estimation create post-selection uncertainty (21). No independent validation, formal multiplicity correction, or *a priori* sample-size/power calculation was performed. Disease-specific analyses were exploratory and crude, with the set of disease categories varying by outcome.

Online recruitment could under-represent the oldest-old, frail adults, people with cognitive impairment, and those with poor digital literacy or limited smartphone/internet access. Absolute fall and recurrent-fall prevalence could plausibly have been underestimated if these groups had greater fall vulnerability (24, 35). The direction of bias in exposure–outcome association estimates cannot be reliably predicted because participation may have been related to both health status and fall vulnerability; its magnitude could not be quantified without nonparticipant-level information. In addition, 35.1% of participants were aged 60–65 and all came from Neijiang, Sichuan. Regional differences in age structure, socioeconomic conditions, healthcare and digital access, housing, physical activity patterns, the built environment, community infrastructure, and environmental safety limit generalizability (16, 29, 35). Income, activity-related, and environmental associations may be particularly context-dependent. Prospective studies across geographically and socioeconomically diverse centers are needed.

## 5 Conclusion

In this cross-sectional study of community-dwelling older adults in Neijiang, fall-related characteristics differed across chronic disease count groups. The clearest descriptive gradient was observed for recurrent falls, while fall occurrence was not strictly monotonic. Timing showed differences as a morning/non-morning contrast, although the full five-category distribution did not show clear overall evidence of heterogeneity. Exploratory post-LASSO analyses described functional, medication-related, sensory, psychological, socioeconomic, and environmental correlates of recurrent falls and non-morning timing. These findings are hypothesis-generating, do not establish temporal or causal relationships, and require confirmation in prospective, multicenter studies.

## Data Availability

The raw data supporting the conclusions of this article will be made available by the authors, without undue reservation.
