# Supplementary material for: Chronic disease count and fall-related characteristics among community-dwelling older adults: a cross-sectional study of recurrent falls and fall timing
Source: Front Public Health. 2026 Oct 12;14:1882689. doi: 10.3389/fpubh.2026.1882689 (PMC13696193; doi:10.3389/fpubh.2026.1882689)
Supplement: Supplementary file 1 [file Table_1.docx]

**Supplementary Material**

**Supplementary Table 1** Additional functional, environmental, and care-related characteristics by fall status

|  | **Fall group** | **Non-fall group** |  |
| --- | --- | --- | --- |
| **Items** | **n = 303** | **n = 307** | **P** |
| **Frailty** |  |  | <0.001 |
| None | 110 (36.3%) | 172 (56.0%) |  |
| Mild | 161 (53.1%) | 124 (40.4%) |  |
| Severe | 32 (10.6%) | 11 (3.6%) |  |
| **Lower limb weakness** |  |  | <0.001 |
| Yes | 213 (70.3%) | 173 (56.4%) |  |
| No | 90 (29.7%) | 134 (43.6%) |  |
| **Balance ability** |  |  | 0.007 |
| Good | 112 (37.0%) | 142 (46.3%) |  |
| Fair | 149 (49.2%) | 143 (46.6%) |  |
| Poor | 42 (13.9%) | 22 (7.2%) |  |
| **Sleep insufficiency** |  |  | <0.001 |
| None | 107 (35.3%) | 148 (48.2%) |  |
| Occasional | 129 (42.6%) | 129 (42.0%) |  |
| Frequent | 67 (22.1%) | 30 (9.8%) |  |
| **Nighttime toileting** |  |  | <0.001 |
| None | 71 (23.4%) | 68 (22.1%) |  |
| Occasional | 132 (43.6%) | 180 (58.6%) |  |
| Frequent | 100 (33.0%) | 59 (19.2%) |  |
| **Need for assistance in ADL** |  |  | <0.001 |
| None | 166 (54.8%) | 241 (78.5%) |  |
| Occasional | 99 (32.7%) | 57 (18.6%) |  |
| Frequent | 38 (12.5%) | 9 (2.9%) |  |
| **Shoes suitability** |  |  | <0.001 |
| Suitable | 150 (49.5%) | 216 (70.4%) |  |
| Unsuitable | 146 (48.2%) | 87 (28.3%) |  |
| Other/unknown | 7 (2.3%) | 4 (1.3%) |  |
| **Bed height suitability** |  |  | <0.001 |
| Suitable | 238 (78.5%) | 279 (90.9%) |  |
| Unsuitable | 65 (21.5%) | 28 (9.1%) |  |
| **Use of walking aid** |  |  | <0.001 |
| Yes | 102 (33.7%) | 36 (11.7%) |  |
| No | 201 (66.3%) | 271 (88.3%) |  |
| **Intravenous infusion access** |  |  | <0.001 |
| Yes | 81 (26.7%) | 22 (7.2%) |  |
| No | 222 (73.3%) | 285 (92.8%) |  |
| **Insufficient room lighting** |  |  | 0.010 |
| Yes | 77 (25.4%) | 52 (16.9%) |  |
| No | 226 (74.6%) | 255 (83.1%) |  |
| **Door threshold at home** |  |  | 0.009 |
| Yes | 114 (37.6%) | 85 (27.7%) |  |
| No | 189 (62.4%) | 222 (72.3%) |  |
| **Slippery bathroom floor** |  |  | <0.001 |
| Yes | 102 (33.7%) | 63 (20.5%) |  |
| No | 201 (66.3%) | 244 (79.5%) |  |
| **Community handrail availability** |  |  | 0.825 |
| Sufficient | 134 (44.2%) | 129 (42.0%) |  |
| Basic | 89 (29.4%) | 91 (29.6%) |  |
| Insufficient | 80 (26.4%) | 87 (28.3%) |  |

Note: Values are n (%); Pearson chi-square tests without continuity correction compare fallers (n = 303) and non-fallers (n = 307). All measures are self-reported.

**Supplementary Table 2** Univariable associations with recurrent falls

| **Items** | **OR (95% CI)** | **P** |
| --- | --- | --- |
| **Sex** |  |  |
| Male | Reference |  |
| Female | 1.59 (0.99–2.55) | 0.057 |
| **Age group** |  |  |
| 60–65 | Reference |  |
| 66–70 | 1.43 (0.67–3.09) | 0.358 |
| 71–75 | 1.19 (0.64–2.22) | 0.576 |
| >75 | 2.20 (1.27–3.81) | 0.005 |
| **Marital status** |  |  |
| Married | Reference |  |
| Others | 1.59 (0.92–2.73) | 0.093 |
| **Education level** |  |  |
| Primary school or below | Reference |  |
| Junior high school | 0.69 (0.41–1.18) | 0.18 |
| High school or above | 0.89 (0.50–1.60) | 0.705 |
| **Living arrangement** |  |  |
| Living alone | Reference |  |
| Living with spouse | 1.05 (0.57–1.95) | 0.866 |
| Living with children | 1.44 (0.84–2.49) | 0.187 |
| **Monthly income (CNY)** |  |  |
| <2000 | Reference |  |
| 2000–4000 | 0.54 (0.32–0.92) | 0.025 |
| 4000–6000 | 0.83 (0.44–1.56) | 0.562 |
| ≥6000 | 0.31 (0.13–0.76) | 0.01 |
| **Medical insurance** |  |  |
| Yes | Reference |  |
| No | 1.73 (0.95–3.12) | 0.071 |
| **Smoking** |  |  |
| No | Reference |  |
| Yes | 0.98 (0.61–1.58) | 0.948 |
| **Alcohol consumption** |  |  |
| No | Reference |  |
| Yes | 1.06 (0.67–1.68) | 0.795 |
| **Physical activity** |  |  |
| No | Reference |  |
| Yes | 0.83 (0.53–1.28) | 0.394 |
| **Hypertension** |  |  |
| No | Reference |  |
| Yes | 1.60 (1.03–2.47) | 0.036 |
| **Diabetes** |  |  |
| No | Reference |  |
| Yes | 1.54 (0.95–2.49) | 0.082 |
| **Osteoarticular disease/osteoporosis** |  |  |
| No | Reference |  |
| Yes | 1.99 (1.27–3.10) | 0.002 |
| **Cardiovascular and cerebrovascular diseases** |  |  |
| No | Reference |  |
| Yes | 2.39 (1.50–3.82) | <0.001 |
| **Multimorbidity** |  |  |
| No | Reference |  |
| Yes | 2.12 (1.36–3.29) | <0.001 |
| **Chronic disease count** |  |  |
| 0 | Reference |  |
| 1 | 1.41 (0.71–2.80) | 0.333 |
| 2 | 1.87 (0.88–3.98) | 0.103 |
| 3 | 2.33 (1.04–5.20) | 0.039 |
| ≥4 | 5.62 (2.51–12.57) | <0.001 |
| **Medication use** |  |  |
| No | Reference |  |
| Yes | 0.49 (0.28–0.86) | 0.013 |
| **CNS depressant medications** |  |  |
| No | Reference |  |
| Yes | 1.85 (0.76–4.47) | 0.174 |
| **Number of medications** |  |  |
| 0 | Reference |  |
| 1 | 1.66 (0.91–3.02) | 0.097 |
| 2 | 1.93 (0.97–3.83) | 0.061 |
| ≥3 | 5.65 (2.54–12.56) | <0.001 |
| **Frailty** |  |  |
| None | Reference |  |
| Mild | 5.32 (2.90–9.76) | <0.001 |
| Severe | 16.65 (7.44–37.23) | <0.001 |
| **Lower limb weakness** |  |  |
| No | Reference |  |
| Yes | 5.47 (2.85–10.50) | <0.001 |
| **Balance ability** |  |  |
| Good | Reference |  |
| Fair | 3.61 (2.01–6.46) | <0.001 |
| Poor | 8.34 (4.07–17.13) | <0.001 |
| **Visual decline** |  |  |
| None | Reference |  |
| Mild | 2.88 (1.50–5.53) | 0.001 |
| Severe | 5.22 (2.50–10.87) | <0.001 |
| **Hearing decline** |  |  |
| None | Reference |  |
| Mild | 2.59 (1.51–4.46) | <0.001 |
| Severe | 6.03 (3.23–11.26) | <0.001 |
| **Dizziness** |  |  |
| None | Reference |  |
| Occasional | 3.37 (1.88–6.04) | <0.001 |
| Frequent | 10.73 (5.17–22.26) | <0.001 |
| **Sleep insufficiency** |  |  |
| None | Reference |  |
| Occasional | 1.87 (1.10–3.19) | 0.022 |
| Frequent | 4.31 (2.36–7.87) | <0.001 |
| **Attention impairment** |  |  |
| None | Reference |  |
| Occasional | 2.06 (1.23–3.46) | 0.006 |
| Frequent | 6.28 (3.15–12.52) | <0.001 |
| **Nighttime toileting** |  |  |
| None | Reference |  |
| Occasional | 1.84 (0.89–3.81) | 0.099 |
| Frequent | 5.41 (2.61–11.21) | <0.001 |
| **Need for assistance in daily activities** |  |  |
| None | Reference |  |
| Occasional | 2.73 (1.65–4.50) | <0.001 |
| Frequent | 8.30 (4.29–16.09) | <0.001 |
| **Shoes suitability** |  |  |
| Suitable | Reference |  |
| Unsuitable | 2.92 (1.84–4.63) | <0.001 |
| Other/unknown | 11.35 (3.29–39.10) | <0.001 |
| **Bed height suitability** |  |  |
| Suitable | Reference |  |
| Unsuitable | 3.25 (1.96–5.40) | <0.001 |
| **Surrounding environment** |  |  |
| Clean | Reference |  |
| Slightly cluttered | 2.29 (1.44–3.66) | <0.001 |
| Severely cluttered | 3.42 (1.52–7.70) | 0.003 |
| **Use of walking aid** |  |  |
| No | Reference |  |
| Yes | 5.56 (3.50–8.83) | <0.001 |
| **Intravenous infusion access** |  |  |
| No | Reference |  |
| Yes | 3.54 (2.17–5.77) | <0.001 |
| **Insufficient room lighting** |  |  |
| No | Reference |  |
| Yes | 2.72 (1.70–4.35) | <0.001 |
| **Familiarity with community environment** |  |  |
| Yes | Reference |  |
| No | 1.64 (1.04–2.59) | 0.032 |
| **Door threshold at home** |  |  |
| No | Reference |  |
| Yes | 2.40 (1.54–3.74) | <0.001 |
| **Slippery bathroom floor** |  |  |
| No | Reference |  |
| Yes | 3.39 (2.16–5.32) | <0.001 |
| **Community handrail availability** |  |  |
| Sufficient | Reference |  |
| Basic | 1.89 (1.09–3.28) | 0.024 |
| Insufficient | 2.49 (1.45–4.27) | <0.001 |
| **Anxiety symptoms** |  |  |
| No | Reference |  |
| Yes | 4.00 (2.55–6.28) | <0.001 |
| **Anxiety level** |  |  |
| None | Reference |  |
| Mild | 3.37 (2.08–5.45) | <0.001 |
| Moderate-to-severe | 8.64 (3.90–19.14) | <0.001 |
| **Depressive symptoms** |  |  |
| No | Reference |  |
| Yes | 2.70 (1.71–4.26) | <0.001 |
| **Depression level** |  |  |
| None | Reference |  |
| Mild | 1.92 (1.15–3.21) | 0.012 |
| Moderate-to-severe | 5.43 (3.01–9.80) | <0.001 |
| **GAD-7 score** | 1.21 (1.14–1.28) | <0.001 |
| **PHQ-9 score** | 1.15 (1.10–1.21) | <0.001 |

**Note:** Recurrent falls were defined as ≥2 falls versus 0–1 falls among all 610 participants. Reference categories are indicated in the table. Multimorbidity was defined as a chronic disease count ≥2 versus 0–1. GAD-7 and PHQ-9 scores of 0–4, 5–9, and ≥10 denote none/minimal, mild, and moderate-to-severe symptoms, respectively; binary anxiety and depressive symptoms were defined using a cutoff of ≥5. ORs are reported with Wald 95% CIs. All analyses are exploratory.

**Supplementary Table 3** Complete exploratory post-LASSO association model for recurrent falls

| **Items** | **OR (95% CI)** | **P** |
| --- | --- | --- |
| **Use of walking aid** |  |  |
| Yes | Reference |  |
| No | 0.36 (0.19–0.69) | 0.002 |
| **Dizziness** |  |  |
| None | Reference |  |
| Occasional | 1.46 (0.72–2.99) | 0.294 |
| Frequent | 4.09 (1.62–10.32) | 0.003 |
| **Lower limb weakness** |  |  |
| Yes | Reference |  |
| No | 0.47 (0.23–0.94) | 0.033 |
| **Slippery bathroom floor** |  |  |
| Yes | Reference |  |
| No | 0.58 (0.34–0.98) | 0.041 |
| **Nighttime toileting** |  |  |
| None | Reference |  |
| Occasional | 0.75 (0.30–1.89) | 0.544 |
| Frequent | 1.28 (0.50–3.30) | 0.607 |
| **Anxiety symptoms** |  |  |
| No | Reference |  |
| Yes | 1.41 (1.04–1.90) | 0.025 |
| **Frailty** |  |  |
| None | Reference |  |
| Mild | 2.61 (1.24–5.49) | 0.012 |
| Severe | 3.41 (1.23–9.44) | 0.018 |
| **Monthly income** |  |  |
| <2000 CNY | Reference |  |
| 2000–4000 CNY | 0.62 (0.32–1.18) | 0.146 |
| 4000–6000 CNY | 0.73 (0.34–1.60) | 0.438 |
| ≥6000 CNY | 0.25 (0.09–0.67) | 0.006 |
| **Intravenous infusion access** |  |  |
| Yes | Reference |  |
| No | 0.67 (0.36–1.28) | 0.226 |
| **Need for assistance in daily activities** |  |  |
| None | Reference |  |
| Occasional | 0.65 (0.31–1.35) | 0.249 |
| Frequent | 1.04 (0.41–2.67) | 0.931 |
| **Hearing decline** |  |  |
| None | Reference |  |
| Mild | 1.31 (0.68–2.55) | 0.419 |
| Severe | 1.51 (0.67–3.39) | 0.323 |
| **Bed height suitability** |  |  |
| Suitable | Reference |  |
| Unsuitable | 1.36 (0.68–2.73) | 0.38 |
| **Chronic disease count** |  |  |
| 0 | Reference |  |
| 1 | 0.58 (0.25–1.37) | 0.215 |
| 2 | 0.75 (0.28–2.02) | 0.571 |
| 3 | 0.52 (0.17–1.63) | 0.261 |
| ≥4 | 0.56 (0.14–2.15) | 0.396 |
| **Shoes suitability** |  |  |
| Suitable | Reference |  |
| Unsuitable | 0.99 (0.53–1.88) | 0.986 |
| Other/unknown | 1.86 (0.32–10.75) | 0.488 |
| **Cardiovascular and cerebrovascular diseases** |  |  |
| Yes | Reference |  |
| No | 0.71 (0.31–1.61) | 0.406 |

**Note:** The model included 15 variables and 26 nonreference coefficients; 96 of 610 participants had recurrent falls. Because variable selection and estimation used the same dataset, ORs, 95% CIs, and P values are exploratory and should not be interpreted as confirmatory inference.

**Supplementary Table 4** Univariable associations with non-morning timing among fallers

| **Items** | **OR (95% CI)** | **P** |
| --- | --- | --- |
| Sex |  |  |
| Male | Reference |  |
| Female | 1.04 (0.63–1.70) | 0.885 |
| Age group |  |  |
| 60–65 | Reference |  |
| 66–70 | 1.51 (0.66–3.45) | 0.331 |
| 71–75 | 1.75 (0.93–3.28) | 0.081 |
| >75 | 1.80 (1.00–3.23) | 0.05 |
| Marital status |  |  |
| Married | Reference |  |
| Others | 1.86 (0.90–3.85) | 0.093 |
| Education level |  |  |
| Primary school or below | Reference |  |
| Junior high school | 0.87 (0.51–1.48) | 0.599 |
| High school or above | 1.44 (0.72–2.89) | 0.299 |
| Living arrangement |  |  |
| Living alone | Reference |  |
| Living with spouse | 2.06 (1.05–4.06) | 0.036 |
| Living with children | 1.15 (0.67–2.00) | 0.609 |
| Monthly income (CNY) |  |  |
| <2000 | Reference |  |
| 2000–4000 | 1.93 (1.06–3.50) | 0.031 |
| 4000–6000 | 1.97 (0.94–4.13) | 0.071 |
| ≥6000 | 2.40 (0.97–5.95) | 0.058 |
| Medical insurance |  |  |
| Yes | Reference |  |
| No | 1.58 (0.73–3.39) | 0.246 |
| Smoking |  |  |
| No | Reference |  |
| Yes | 2.34 (1.31–4.17) | 0.004 |
| Alcohol consumption |  |  |
| No | Reference |  |
| Yes | 2.05 (1.20–3.51) | 0.009 |
| Physical activity |  |  |
| No | Reference |  |
| Yes | 0.46 (0.29–0.75) | 0.002 |
| Hypertension |  |  |
| No | Reference |  |
| Yes | 1.65 (1.01–2.69) | 0.047 |
| Diabetes |  |  |
| No | Reference |  |
| Yes | 1.89 (1.07–3.32) | 0.028 |
| Osteoarticular disease/osteoporosis |  |  |
| No | Reference |  |
| Yes | 1.90 (1.14–3.19) | 0.015 |
| Cardiovascular and cerebrovascular diseases |  |  |
| No | Reference |  |
| Yes | 1.42 (0.81–2.47) | 0.222 |
| Multimorbidity |  |  |
| No | Reference |  |
| Yes | 2.11 (1.29–3.44) | 0.003 |
| Chronic disease count |  |  |
| 0 | Reference |  |
| 1 | 1.49 (0.81–2.76) | 0.201 |
| 2 | 2.77 (1.29–5.94) | 0.009 |
| 3 | 2.18 (0.98–4.85) | 0.055 |
| ≥4 | 3.19 (1.28–7.97) | 0.013 |
| Medication use |  |  |
| No | Reference |  |
| Yes | 0.60 (0.36–1.00) | 0.052 |
| CNS depressant medications |  |  |
| No | Reference |  |
| Yes | 1.12 (0.44–2.86) | 0.819 |
| Number of medications |  |  |
| 0 | Reference |  |
| 1 | 1.25 (0.71–2.18) | 0.442 |
| 2 | 3.30 (1.54–7.06) | 0.002 |
| ≥3 | 1.66 (0.72–3.82) | 0.237 |
| Frailty |  |  |
| None | Reference |  |
| Mild | 1.68 (1.02–2.78) | 0.041 |
| Severe | 2.87 (1.14–7.19) | 0.025 |
| Lower limb weakness |  |  |
| No | Reference |  |
| Yes | 1.82 (1.10–3.02) | 0.02 |
| Balance ability |  |  |
| Good | Reference |  |
| Fair | 3.37 (2.00–5.68) | <0.001 |
| Poor | 5.08 (2.16–11.96) | <0.001 |
| Visual decline |  |  |
| None | Reference |  |
| Mild | 2.94 (1.71–5.06) | <0.001 |
| Severe | 4.82 (2.25–10.32) | <0.001 |
| Hearing decline |  |  |
| None | Reference |  |
| Mild | 5.83 (3.32–10.23) | <0.001 |
| Severe | 7.57 (3.29–17.45) | <0.001 |
| Dizziness |  |  |
| None | Reference |  |
| Occasional | 1.70 (1.02–2.82) | 0.042 |
| Frequent | 2.84 (1.23–6.56) | 0.014 |
| Sleep insufficiency |  |  |
| None | Reference |  |
| Occasional | 2.35 (1.37–4.04) | 0.002 |
| Frequent | 1.99 (1.05–3.79) | 0.035 |
| Attention impairment |  |  |
| None | Reference |  |
| Occasional | 3.86 (2.27–6.58) | <0.001 |
| Frequent | 2.19 (0.99–4.84) | 0.054 |
| Nighttime toileting |  |  |
| None | Reference |  |
| Occasional | 2.01 (1.11–3.63) | 0.02 |
| Frequent | 2.27 (1.21–4.27) | 0.011 |
| Need for assistance in daily activities |  |  |
| None | Reference |  |
| Occasional | 2.10 (1.22–3.61) | 0.007 |
| Frequent | 1.83 (0.85–3.94) | 0.12 |
| Shoes suitability |  |  |
| Suitable | Reference |  |
| Unsuitable | 2.75 (1.68–4.50) | <0.001 |
| Other/unknown | 5.39 (0.63–45.88) | 0.123 |
| Bed height suitability |  |  |
| Suitable | Reference |  |
| Unsuitable | 2.38 (1.25–4.54) | 0.009 |
| Surrounding environment |  |  |
| Clean | Reference |  |
| Slightly cluttered | 1.69 (1.03–2.77) | 0.037 |
| Severely cluttered | 4.75 (1.35–16.73) | 0.015 |
| Use of walking aid |  |  |
| No | Reference |  |
| Yes | 1.42 (0.86–2.37) | 0.175 |
| Intravenous infusion access |  |  |
| No | Reference |  |
| Yes | 0.80 (0.47–1.35) | 0.397 |
| Insufficient room lighting |  |  |
| No | Reference |  |
| Yes | 2.89 (1.55–5.39) | <0.001 |
| Familiarity with community environment |  |  |
| Yes | Reference |  |
| No | 3.41 (1.90–6.09) | <0.001 |
| Door threshold at home |  |  |
| No | Reference |  |
| Yes | 1.61 (0.98–2.66) | 0.06 |
| Slippery bathroom floor |  |  |
| No | Reference |  |
| Yes | 2.72 (1.57–4.71) | <0.001 |
| Community handrail availability |  |  |
| Sufficient | Reference |  |
| Basic | 3.27 (1.78–6.02) | <0.001 |
| Insufficient | 1.84 (1.03–3.28) | 0.038 |
| Anxiety symptoms |  |  |
| No | Reference |  |
| Yes | 2.52 (1.50–4.23) | <0.001 |
| Anxiety level |  |  |
| None | Reference |  |
| Mild | 2.05 (1.19–3.52) | 0.01 |
| Moderate-to-severe | 8.41 (1.92–36.78) | 0.005 |
| Depressive symptoms |  |  |
| No | Reference |  |
| Yes | 2.76 (1.68–4.51) | <0.001 |
| Depression level |  |  |
| None | Reference |  |
| Mild | 2.57 (1.48–4.46) | <0.001 |
| Moderate-to-severe | 3.22 (1.50–6.92) | 0.003 |
| GAD-7 score | 1.18 (1.10–1.27) | <0.001 |
| PHQ-9 score | 1.13 (1.06–1.20) | <0.001 |

**Note:** Outcome was non-morning versus morning timing among 303 fallers (195 vs 108). Chronic disease count used 0 as reference; multimorbidity was defined as ≥2 versus 0–1 diseases, whereas main Table 6 used 0–1 as the pooled reference. GAD-7/PHQ-9 categories were 0–4, 5–9, and ≥10; binary symptoms used ≥5. The physical-activity OR was 0.46 in this univariable analysis versus 1.88 in the post-LASSO model. No formal multiplicity adjustment was applied.

**Supplementary Table 5** Complete exploratory post-LASSO association model for non-morning timing among fallers

| **Items** | **OR (95% CI)** | **P** |
| --- | --- | --- |
| **Chronic disease count** |  |  |
| 0 | Reference |  |
| 1 | 1.08 (0.52–2.23) | 0.840 |
| 2 | 1.31 (0.52–3.29) | 0.563 |
| 3 | 1.42 (0.51–3.95) | 0.501 |
| ≥4 | 1.56 (0.47–5.19) | 0.469 |
| **Number of medications** |  |  |
| 0 | Reference |  |
| 1 | 1.34 (0.72–2.49) | 0.356 |
| 2 | 2.18 (1.08–4.42) | 0.030 |
| ≥3 | 2.76 (1.13–6.72) | 0.026 |
| **Frailty** |  |  |
| None | Reference |  |
| Mild | 1.29 (0.68–2.45) | 0.435 |
| Severe | 1.61 (0.55–4.72) | 0.383 |
| **Balance ability** |  |  |
| Good | Reference |  |
| Fair | 2.05 (1.12–3.77) | 0.020 |
| Poor | 3.48 (1.31–9.25) | 0.012 |
| **Dizziness** |  |  |
| None | Reference |  |
| Occasional | 1.76 (1.01–3.07) | 0.046 |
| Frequent | 2.89 (1.12–7.46) | 0.028 |
| **Hearing decline** |  |  |
| None | Reference |  |
| Mild | 3.96 (2.08–7.55) | <0.001 |
| Severe | 5.72 (2.18–15.01) | <0.001 |
| **Attention impairment** |  |  |
| None | Reference |  |
| Occasional | 1.48 (0.78–2.81) | 0.230 |
| Frequent | 1.62 (0.58–4.51) | 0.356 |
| **Physical activity** |  |  |
| No | Reference |  |
| Yes | 1.88 (1.08–3.27) | 0.025 |
| **Familiarity with community environment** | |  |
| Yes | Reference |  |
| No | 1.54 (0.79–3.02) | 0.208 |
| **Community handrail availability** |  |  |
| Sufficient | Reference |  |
| Basic | 1.83 (1.02–3.30) | 0.043 |
| Insufficient | 2.41 (1.19–4.89) | 0.015 |

**Note:** The model included 10 variables and 21 nonreference coefficients. Because variable selection and estimation used the same dataset, ORs, 95% CIs, and P values are exploratory and should not be interpreted as confirmatory inference. The physical-activity estimate was opposite in direction to the univariable estimate; neither analysis establishes a harmful effect of physical activity.

**Supplementary Table 6** Exploratory multidomain attenuation model

| **Chronic disease count** | **Crude OR (95% CI)** | **Model 1 OR (95% CI)** | **Model 2 OR (95% CI)** |
| --- | --- | --- | --- |
| **Fall occurrence** |  |  |  |
| 0 chronic diseases | Reference | Reference | Reference |
| 1 chronic disease | 0.65 (0.42–1.00) | 0.61 (0.39–0.96) | 0.56 (0.31–1.04) |
| 2 chronic diseases | 0.81 (0.50–1.34) | 0.92 (0.54–1.56) | 0.82 (0.38–1.77) |
| 3 chronic diseases | 1.16 (0.66–2.04) | 1.23 (0.68–2.25) | 0.71 (0.29–1.70) |
| ≥4 chronic diseases | 1.94 (0.98–3.84) | 1.85 (0.90–3.84) | 0.68 (0.23–1.97) |
| *P* for trend | 0.027 | 0.018 | 0.799 |
| **Recurrent falls** |  |  |  |
| 0 chronic diseases | Reference | Reference | Reference |
| 1 chronic disease | 1.41 (0.71–2.80) | 1.27 (0.63–2.59) | 0.59 (0.23–1.50) |
| 2 chronic diseases | 1.87 (0.88–3.98) | 1.93 (0.87–4.25) | 0.84 (0.28–2.53) |
| 3 chronic diseases | 2.33 (1.04–5.20) | 2.18 (0.94–5.06) | 0.65 (0.20–2.16) |
| ≥4 chronic diseases | 5.62 (2.51–12.57) | 4.85 (2.06–11.46) | 1.03 (0.29–3.71) |
| *P* for trend | <0.001 | <0.001 | 0.676 |
| **Non-morning timing** |  |  |  |
| 0–1 chronic disease | Reference | Reference | Reference |
| 2 chronic diseases | 2.20 (1.12–4.34) | 1.56 (0.74–3.29) | 1.20 (0.56–2.59) |
| 3 chronic diseases | 1.74 (0.85–3.56) | 1.25 (0.56–2.75) | 1.16 (0.51–2.62) |
| ≥4 chronic diseases | 2.54 (1.09–5.92) | 1.90 (0.76–4.77) | 1.48 (0.57–3.81) |
| *P* for trend | 0.008 | 0.156 | 0.417 |

**Note:** Model 1 adjusts for sex, age group, smoking, alcohol consumption, and physical activity. Model 2 additionally includes medication-related, functional, sensory, psychological, and environmental variables. Reference groups are 0 chronic diseases for fall occurrence and recurrent falls and 0–1 chronic diseases for non-morning timing. Trend scores are 0–4, 0–4, and 0–3, respectively. Model 2 is exploratory; attenuation does not establish mediation, absence of association, or an independent causal effect. OR, odds ratio; CI, confidence interval.

**Supplementary Table 7.** Outcome counts and nominal model dimensions

| **Model** | **N** | **Outcome 1 / 0** | **Nominal slopes** | **Outcome 1 per slope** | **Smaller group per slope** |
| --- | --- | --- | --- | --- | --- |
| Model 1 recurrent | 610 | 96 / 514 | 11 | 8.73 | 8.73 |
| Model 1 timing | 303 | 195 / 108 | 10 | 19.50 | 10.80 |
| Multidomain recurrent | 610 | 96 / 514 | ≈50 | ≈1.92 | ≈1.92 |
| Multidomain timing | 303 | 195 / 108 | ≈49 | ≈3.98 | ≈2.20 |
| Post-LASSO recurrent | 610 | 96 / 514 | 26 | 3.69 | 3.69 |
| Post-LASSO timing | 303 | 195 / 108 | 21 | 9.29 | 5.14 |

**Note:** Counts exclude the intercept and represent nominal model dimensions rather than effective fitted ranks. Outcome 1 denotes recurrent falls or non-morning timing, as applicable. Sparse cells, correlated variables, and aliased terms may affect the effective fitted dimension.

**Supplementary Table 8.** Nominal parameter composition of the recurrent-fall multidomain model

| **Parameter block** | **Nominal slopes** |
| --- | --- |
| Chronic disease count (0 reference) | 4 |
| Model 1: sex 1, age 3, smoking 1, alcohol 1, activity 1 | 7 |
| Medication: use 1, CNS depressants 1, count 3 | 5 |
| Functional/sensory: frailty 2, weakness 1, balance 2, vision 2, hearing 2, dizziness 2, sleep 2, attention 2, toileting 2, ADL assistance 2 | 19 |
| Environmental: footwear 2, bed height 1, surroundings 2, walking aids 1, infusion access 1, lighting 1, familiarity 1, threshold 1, slippery floor 1, handrails 2 | 13 |
| Psychological: anxiety symptoms 1, depressive symptoms 1 | 2 |
| Total | 50 |

**Note:** The recurrent-fall multidomain model included 50 nominal slopes. The pooled-reference timing model included approximately 49 because chronic disease count contributed three rather than four slopes. Post-LASSO dimensions of 26 and 21 refer to separate refitted models.

**Supplementary Table 9.** Equal-duration sensitivity analysis of morning versus afternoon falls

| **Chronic disease count** | **06:00–<12:00, n (%)** | **12:00–<18:00, n (%)** | **Total** |
| --- | --- | --- | --- |
| 0 | 35 (71.4) | 14 (28.6) | 49 |
| 1 | 38 (54.3) | 32 (45.7) | 70 |
| 2 | 14 (41.2) | 20 (58.8) | 34 |
| 3 | 13 (52.0) | 12 (48.0) | 25 |
| ≥4 | 8 (32.0) | 17 (68.0) | 25 |
| Total | 108 (53.2) | 95 (46.8) | 203 |

**Note:** Percentages are based on the two 6-hour comparison windows; the other 100 of 303 fallers were excluded. Pearson χ²(4) = 13.07, P = 0.011; minimum expected count = 11.70. This exploratory analysis does not account for activity-time exposure or multiple comparisons and does not validate the broader morning/non-morning contrast.

**Supplementary Table 10.** Summary of fall-timing comparison tests

| **Timing comparison** | **χ²** | **df** | **Pearson P** |
| --- | --- | --- | --- |
| Five categories across count groups | 20.582 | 16 | 0.195 |
| Morning versus non-morning | 11.230 | 4 | 0.024 |
| Morning versus afternoon | 13.074 | 4 | 0.011 |

**Note:** The five-category table had 7 of 25 expected counts below 5 (minimum 1.733). A fixed-margin conditional Monte Carlo Pearson test using 200,000 random tables yielded P = 0.196. This test is distinct from a probability-ordering Fisher exact test.

**Supplementary Table 11.** Exploratory associations of individual chronic disease categories with fall-related outcomes

| **Outcome** | **Disease category (Yes versus No)** | **Crude OR (95% CI)** | **Wald P** |
| --- | --- | --- | --- |
| Fall occurrence | Hypertension | 0.89 (0.65–1.23) | 0.493 |
|  | Diabetes | 1.53 (1.05–2.23) | 0.029 |
|  | Osteoarticular disease/osteoporosis | 1.38 (0.98–1.95) | 0.064 |
|  | Cardiovascular and cerebrovascular diseases | 1.35 (0.92–1.97) | 0.125 |
|  | Hyperlipidemia | 0.73 (0.44–1.21) | 0.227 |
|  | Other physician-diagnosed chronic disease | 0.71 (0.46–1.11) | 0.135 |
| Recurrent falls | Hypertension | 1.60 (1.03–2.47) | 0.036 |
|  | Diabetes | 1.54 (0.95–2.49) | 0.082 |
|  | Osteoarticular disease/osteoporosis | 1.99 (1.27–3.10) | 0.002 |
|  | Cardiovascular and cerebrovascular diseases | 2.39 (1.50–3.82) | <0.001 |
| Non-morning timing | Hypertension | 1.65 (1.01–2.69) | 0.047 |
|  | Diabetes | 1.89 (1.07–3.32) | 0.028 |
|  | Osteoarticular disease/osteoporosis | 1.90 (1.14–3.19) | 0.015 |
|  | Cardiovascular and cerebrovascular diseases | 1.42 (0.81–2.47) | 0.222 |

**Note:** All contrasts compare Yes versus No and are crude, unadjusted associations. Fall occurrence was assessed for six descriptive disease categories, whereas recurrent falls and non-morning timing were assessed for hypertension, diabetes, osteoarticular disease/osteoporosis, and combined cardiovascular/cerebrovascular disease. The combined cardiovascular/cerebrovascular indicator does not distinguish its two counted domains. These analyses are exploratory, and no formal multiplicity adjustment was applied.

**Supplementary Table 12.** Disease count and variable definitions

| **Counted disease domain** | **Contribution** |
| --- | --- |
| Hypertension | 0 or 1 |
| Diabetes | 0 or 1 |
| Osteoarticular disease/osteoporosis | 0 or 1; one shared domain |
| Heart disease | 0 or 1 |
| Cerebrovascular disease | 0 or 1 |
| Hyperlipidemia | 0 or 1 |
| Other physician-diagnosed chronic disease | 0 or 1; at most one point |

**Note:** The count is unweighted and does not include severity, duration, complications, treatment intensity, or disease-specific functional impact. Groups are 0, 1, 2, 3, and ≥4, with ≥4 meaning four or more counted categories. Heart disease and cerebrovascular disease are counted separately but displayed together in Table 1 and the disease-specific crude table. A sum of the six combined descriptive indicators is therefore not the seven-domain count.

**Supplementary Table 13.** Crude multimorbidity associations with recurrent falls and non-morning timing

| **Outcome** | **Outcome 1 / 0 in ≥2** | **Outcome 1 / 0 in 0–1** | **Crude OR (95% CI)** | **Wald P** |
| --- | --- | --- | --- | --- |
| Recurrent falls | 53 / 189 | 43 / 325 | 2.12 (1.36–3.29) | <0.001 |
| Non-morning timing | 98 / 35 | 97 / 73 | 2.11 (1.29–3.44) | 0.003 |

**Note:** Multimorbidity was defined as a chronic disease count ≥2 versus 0–1. Recurrent falls were defined as ≥2 falls versus 0–1 falls among all participants; non-morning timing was compared with morning timing among fallers. All estimates are crude and unadjusted. ORs are reported with Wald 95% CIs.

**Supplementary Table 14.** Exploratory LASSO workflow

| **Component** | **Specification** |
| --- | --- |
| Software | R 4.5.1; glmnet |
| Family and input | Binomial; categorical variables dummy coded; inputs standardized |
| Candidate screening | Univariable P < 0.05; 35 recurrent-fall candidates and 34 timing candidates |
| Penalty selection | Cross-validation; λ_1se_ selected for subsequent models |
| Recurrent-fall λ | λ_min_ = 0.006997; λ_1se_ = 0.013102 |
| Timing λ | λ_min_ = 0.007024; λ_1se_ = 0.011655 |
| Figures 4/5 horizontal axes | log(λ); the λ values above are untransformed |
| Post-LASSO refitting | Full factor categories fitted after selection; 15 recurrent-fall variables, 10 timing variables |
| Interpretation | Same-dataset selection and estimation; exploratory association estimates; no independent validation |

**Note:** Refitting full factor categories can yield more nominal coefficients than selected nonzero dummy features; therefore, the post-LASSO dimensions of 26 and 21 differ from the selected feature counts. No bootstrap stability analysis or independent validation was performed, and conventional P values do not account for selection uncertainty.
